# Do We Need a Different Debate About How to Manage Pandemics?

**DOI:** 10.1093/function/zqab075

**Published:** 2022-01-03

**Authors:** Ole H Petersen

**Affiliations:** School of Biosciences, Sir Martin Evans Building, Cardiff University, Wales CF10 3AX, UK

We have now lived for 2 yr with COVID-19, and there is no indication—at the time of writing this editorial—that we are anywhere near the end of this pandemic. We have got used to having to wear masks, work from home, being repeatedly tested, and vaccinated as well as having to endure constantly changing travel restrictions. In spite of all these measures, which have reduced the quality of life throughout the globe, COVID-19 has so far resulted in more than 5 million deaths worldwide ^1^ and has had a very significant effect on world economics. In comparison, the 1918–1920 flu pandemic may have claimed more than 50 million lives, including many healthy individuals in the age group 20–40.^2^ However, in the US for example, more people have already died of COVID-19 (> 800 000) than died of flu in the 1918–1920 pandemic (< 700 000).^1^^,^^2^

The last 2 yr have been an extraordinary period for the Life Sciences. There have been some remarkable achievements within an unusually short time frame and biomedical science progress has been prominently featured, but not always correctly, in virtually all media worldwide with unprecedented prominence. Indeed, some biomedical scientists have become media stars. Scientific advice for government policy has also become more central and been more visible than ever before. However, as the German proverb “Bäume wachsen nicht in den Himmel“ (trees do not grow to the sky) implies, what has sometimes been described as a triumph of biomedical science is not nearly as complete as we might like to think. The impressively fast generation and production of vaccines as well as the effective roll-out of vaccinations in many countries has undoubtedly saved many lives, but has not ended the pandemic. After the initial euphoria, we now realize that the immunity wanes more rapidly than anticipated, ^3^ and that virus variants can render current vaccines much less effective than hoped for. ^4^

In spite of significant medical progress with regard to prevention, diagnosis, and treatment, many feel that in the current pandemic we are staggering from one crisis to another with no clear plan for the future. The public debate about the measures to be taken in the defence against SARS-CoV-2 has been primitive and the division of opinions has largely followed political divisions. So-called right-wing groups oppose vaccinations and any restrictions of individual movements and contacts, whereas left-of-centre opinion favours vaccinations, and in many cases mandatory vaccinations, as well as even severe restrictions in the freedom of movement and association of individuals.

Have the measures taken against SARS-CoV-2 been effective? There are enormous differences in the way different governments have reacted to the crisis. At one extreme, China introduced, and continues to use, very severe quarantines, in some cases closing down completely even very large cities, although mostly for relatively short periods. Movements in and out of China have also been drastically restricted, in some respects effectively closing down contact with other countries. These measures have been extremely effective and in spite of China's vast population, there have been less than 5000 COVID-related deaths. ^1^ In contrast, the so-called western world has introduced much more limited restrictions to travel and meetings, to a large extent now relying on massive vaccination programmes. While many western governments claim that they are “following the science” this is in reality frequently not the case, as some of the scientific advice given has proven to be politically unacceptable. As a result, the number of deaths in relation to population sizes are generally orders of magnitude higher in the western world than in China and many other Asian countries. ^1^

In a remarkably frank and interesting account of the “biopolitics” in Beijing during the COVID pandemic, by a scholar from the University of International Business and Economics in Beijing, ^5^ it is admitted that “Social control plays a vital role in the biopolitical practices implemented in Beijing during the pandemic, with coercive measures imposed by an authoritarian government.” and “The tight social control called for by the epidemiological nature of Covid-19 can be implemented most effectively by an authoritarian government..” It is, thus made crystal clear that the remarkably effective measures taken in China to defend the population against SARS-CoV-2 can only be carried out in a country ruled by a dictatorial government.

Therefore, Chinese biopolitics cannot be implemented in pluralistic western societies which furthermore, unlike many Asian democracies, do not have a current culture of general obedience to those elected to govern. In Europe and the US, all Covid-related measures implemented, or even just recommended, have been contested, both in parliamentary assemblies, in the media, and on the streets. There have also on many occasions been public disagreements between scientific advisors and between advisors and government ministers about the required measures to be taken. In many countries, the precise restrictive measures taken in the fight against COVID-19 often seem arbitrary. In the UK, for example, different jurisdictions (England, Scotland, Wales, and Northern Ireland) have repeatedly issued different regulations and advice.

A state of uncertainty and confusion was inevitable at the start of the pandemic, and therefore generally acceptable, but as the crisis continues with no clear end point in sight, there is a need for both a meaningful debate about the future and the emergence of credible intellectual leadership in guiding us into the future. We have to get away from the primitive division between those who do not want any regulations and those who feel the need to regulate all aspects of interpersonal contacts. We need scientific advisors to be very clear about what they know and what they do not know, and to carefully explain the specific rationale for their recommendations. It would be good if the media could stop the prevalent general scaremongering, frequently with strongly nationalistic overtones, and focus on accurate reporting of the issues rather than endlessly recirculate political disagreements that lead nowhere. We need political leaders to listen to proper evidence-informed advice, but then be honest about the reasons why they do not always follow that advice.

There are enormous gaps in our knowledge about the consequences of almost all aspects of the current crisis and, since the COVID-19 pandemic is unlikely to be the last such event, we need to invest properly in all the relevant branches of science and scholarship that may allow us to do better when the next pandemic arrives. Above all, responsible leaders must admit that this is a global crisis which, like the climate crisis, can only be solved at a global level. Therefore, the United Nations (UN), including the WHO, needs to be significantly strengthened. It is high time to work on long term plans.
